# Virus-Host Coevolution: Common Patterns of Nucleotide Motif Usage in *Flaviviridae* and Their Hosts

**DOI:** 10.1371/journal.pone.0006282

**Published:** 2009-07-20

**Authors:** Francisco P. Lobo, Bruno E. F. Mota, Sérgio D. J. Pena, Vasco Azevedo, Andréa M. Macedo, Andreas Tauch, Carlos R. Machado, Glória R. Franco

**Affiliations:** 1 Departamento de Bioquímica e Imunologia, Universidade Federal de Minas Gerais, Belo Horizonte, Minas Gerais, Brazil; 2 Departamento de Microbiologia, Universidade Federal de Minas Gerais, Belo Horizonte, Minas Gerais, Brazil; 3 Departamento de Biologia Geral, Universidade Federal de Minas Gerais, Belo Horizonte, Minas Gerais, Brazil; 4 Center for Biotechnology (CeBiTec), Bielefeld University, Bielefeld, Germany; Institut Pasteur, France

## Abstract

Virus-host biological interaction is a continuous coevolutionary process involving both host immune system and viral escape mechanisms. *Flaviviridae* family is composed of fast evolving RNA viruses that infects vertebrate (mammals and birds) and/or invertebrate (ticks and mosquitoes) organisms. These host groups are very distinct life forms separated by a long evolutionary time, so lineage-specific anti-viral mechanisms are likely to have evolved. *Flaviviridae* viruses which infect a single host lineage would be subjected to specific host-induced pressures and, therefore, selected by them. In this work we compare the genomic evolutionary patterns of *Flaviviridae* viruses and their hosts in an attempt to uncover coevolutionary processes inducing common features in such disparate groups. Especially, we have analyzed dinucleotide and codon usage patterns in the coding regions of vertebrate and invertebrate organisms as well as in *Flaviviridae* viruses which specifically infect one or both host types. The two host groups possess very distinctive dinucleotide and codon usage patterns. A pronounced CpG under-representation was found in the vertebrate group, possibly induced by the methylation-deamination process, as well as a prominent TpA decrease. The invertebrate group displayed only a TpA frequency reduction bias. *Flaviviridae* viruses mimicked host nucleotide motif usage in a host-specific manner. Vertebrate-infecting viruses possessed under-representation of CpG and TpA, and insect-only viruses displayed only a TpA under-representation bias. Single-host *Flaviviridae* members which persistently infect mammals or insect hosts (*Hepacivirus* and insect-only *Flavivirus*, respectively) were found to posses a codon usage profile more similar to that of their hosts than to related *Flaviviridae*. We demonstrated that vertebrates and mosquitoes genomes are under very distinct lineage-specific constraints, and *Flaviviridae* viruses which specifically infect these lineages appear to be subject to the same evolutionary pressures that shaped their host coding regions, evidencing the lineage-specific coevolutionary processes between the viral and host groups.

## Introduction

Virus-host pair is a unique situation to study coevolutionary processes, as viruses are obligate intracellular parasites and their evolution is inexorably coupled to host biology. The biological interaction of viruses and their hosts is a delicate balance of actions and counteractions between host immune system and virus escape mechanisms. Viruses are unable to replicate without infecting host cells, a process which commonly leads to host fitness decrease [1], [2]. In these circumstances, hosts which develop strategies to avoid or limit virus infection are expected to be favored by natural selection. In accordance with this supposition, a broad range of anti-viral mechanisms are known to occur in practically all living taxa, such as RNAi [3]–[5] in many Eukaryote lineages, pattern recognition receptors [6]–[8] in metazoan and plants and restriction-modification systems in Eubacteria and Archaea [9], [10], to cite a few. In a similar way, viruses which develop mechanisms to evade host natural defenses are expected to be favored as well [11], [12], leading to a sequential counter-adaptation process in both organisms [13]–[15].

*Flaviviridae* virus family comprises small, enveloped, positive-sense, single-stranded RNA viruses which infect vertebrate (mammals and birds) and/or invertebrate (mosquitoes and ticks) hosts. Due to their relatively small genome size and medical and veterinarian importance, several *Flaviviridae* viruses have already had their genomes sequenced [16]. The viral genomes vary from 9 to 13 Kb and contain a single known ORF that codes for a polyprotein, which is processed co- and post-translationally by host and viral proteases into at least 10 functional, individual polypeptides [17]. This family is composed of three known genera (*Flavivirus*, *Hepacivirus* and *Pestivirus*) which vary in their host range, specificity and outcome of infection. *Flavivirus* genus comprises approximately 70 viruses, and is unique among *Flaviviridae* genera since it contains the only arthropod-borne viruses (arboviruses) known in this family, which are transmitted to vertebrate hosts by mosquitoes or ticks via blood feeding. Some of these arboviruses are associated with severe human diseases, such as dengue virus (DENV [18]) and yellow fever virus (YFV [19]). *Flavivirus* members can be classified into four main ecological groups based on known host range and transmission mode [20]. They are: insect-only, non-known arthropod vector (NKV), insect-borne and tick-borne. The insect-only viruses are able to replicate solely in invertebrate cell culture and where isolated from insect cell lines or from field-collected mosquitoes. The known insect-only viruses with complete genome sequences available are the cell-fusing agent virus (CFAV, [21]), the Kamiti river virus (KRV, [22]) and the Culex *Flavivirus* ([23]). Another ecological group found in *Flavivirus* genus is composed of NKV viruses, found thus far infecting only vertebrate hosts. This viral group is paraphyletic and contains at least three distinct lineages: the first is the Tamana Bat Virus (TABV), a very divergent *Flavivirus* member isolated from bats [24]; a second group is closely related to the mosquito-borne arboviruses of YF lineage; and a third group, a sister-group of the tick-borne arboviruses [25]. The two remaining *Flavivirus* groups are arboviruses - a mosquito-borne and a tick-borne. The mosquito-borne group is also of paraphyletic origin and contains two main clades (the YFV and DENV lineages); the tick-borne lineage is apparently a monophyletic group [25]. *Hepacivirus* comprises a genus of mammal-exclusive viruses mainly transmitted by blood contact. Its type species is Hepatitis C virus (HCV), which has been found to cause hepatitis in more than 1% of the world's human population, and several other liver-related diseases such as cirrhosis and liver cancer [26]. Two other *Flaviviridae* species, GB virus-B (GBV-B) [27] and GB virus-C (GBV-C) [28], share extensive gene homology with HCV ([29] and this study), and are currently being considered as *Hepacivirus* genus candidates by the International Committee on Taxonomy of Viruses (ICTV) [30]. *Pestivirus* genus is composed of several species which infect non-human mammals, mainly via oral-fecal or respiratory routes. Currently five viral species are classified as belonging to *Pestivirus* by ICTV [30].

Arbovirus is a non-taxonomic designation for RNA viruses which, in nature, replicate cyclically in vertebrate and arthropod hosts, and are transmitted to vertebrate hosts by haematophagous vectors [31]. An intriguing phenomenon observed in several arboviruses when replicating in vertebrate-invertebrate cycle is their lower evolutionary rates compared to single-host related viruses or to arboviruses replicating solely in one host type in experimental conditions [1], [32], [33]. These observations led to the proposition of a fitness trade-off situation occurring in arboviruses as a consequence of infecting very distinct host types. Alternating replication in vertebrate and arthropod organisms would impose contrasting selective forces, where fitness increase in a given host cell type leads to fitness decrease in the other, generating a scenario of viral sub-optimal adaptation in each host *milieu*. One should expect, under this assumption, a rapid and specific adaptation of arboviruses to individual hosts types when replicating in a single-host system. This was previously observed in several *in vitro* and *in vivo* experiments, where single host infection led to host-specific viral fitness increase and higher mutation rates [34]–[36].

Vertebrate and arthropod lineages diverged between 573 and 656 million years ago [37], a considerable evolutionary time which changed these groups in radically different ways. It is reasonable to suppose that this divergence time was sufficient for completely new anti-viral mechanisms to emerge in a lineage-specific manner, and viruses which specifically infect these groups would be subjected to distinct, host-specific evolutionary pressures. Genomes of eukaryotic RNA viruses and host mRNA molecules coexist in the same cellular compartment (cytoplasm), and viral and host coding regions are expected to share some common compositional features due to constraints induced by factors coded by hosts genomes. In this work, we evaluated the patterns of nucleotide motif usage in vertebrates and mosquitoes species and in *Flaviviridae* viruses which specifically infect one or both host types. As shown below, we found several viral nucleotide usage patterns which are correlated with host-specific patterns, suggesting that host-induced pressures are actively shaping dinucleotide and codon usage patterns in both *Flaviviridae* and hosts coding regions.

## Results and Discussion

The hosts group was divided into vertebrates and invertebrates, and viruses were divided into six groups to reflect their evolutionary history as well as their host specificity and range (see “*Flaviviridae* Phylogenetic Analysis” section below). *Hepacivirus* group and *Pestivirus* genus were considered distinct vertebrate-exclusive groups, and *Flavivirus* genus was further divided into four groups according to phylogenetic relatedness and/or host type, generating the NKV, insect-only, mosquito-borne and tick-borne *Flavivirus* groups. Individual group composition is listed in Table S1.

### Flaviviridae Phylogenetic Analysis

**Figure 1:**
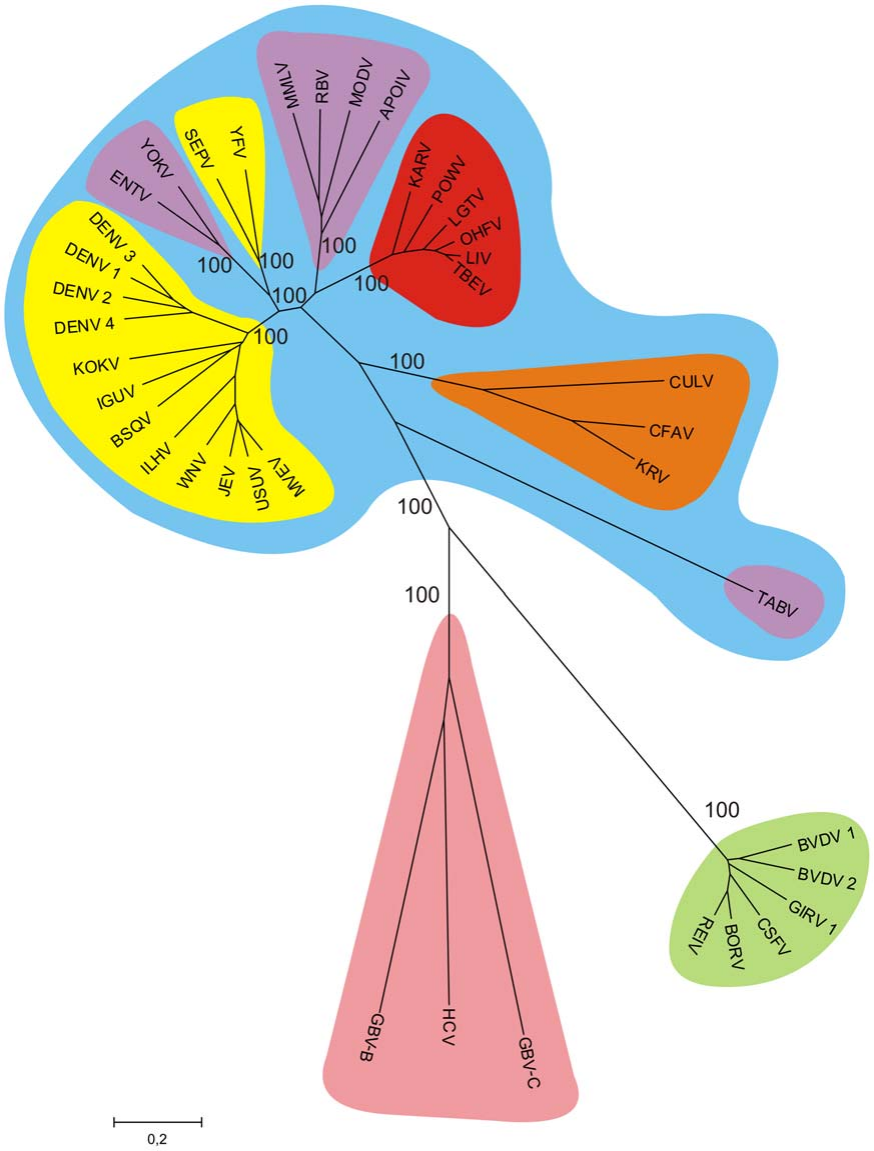
Phylogenetic relationships among *Flaviviridae* viruses. Phylogenetic trees were constructed with whole polyprotein sequences using the Neighbor-Joining method. Bootstrap values were calculated after 1000 replicates, and the consensus tree is shown. *Flavivirus* genus is highlighted in blue, *Pestivirus* is in green and *Hepacivirus* is in salmon. *Flavivirus* genus was further divided to reflect distinct viral host range, with insect-only viruses in orange, NKV in purple, mosquito-borne in yellow and tick-borne in red. Acronyms and names of the viruses are located in the “viral dataset” section.

**Figure 2:**
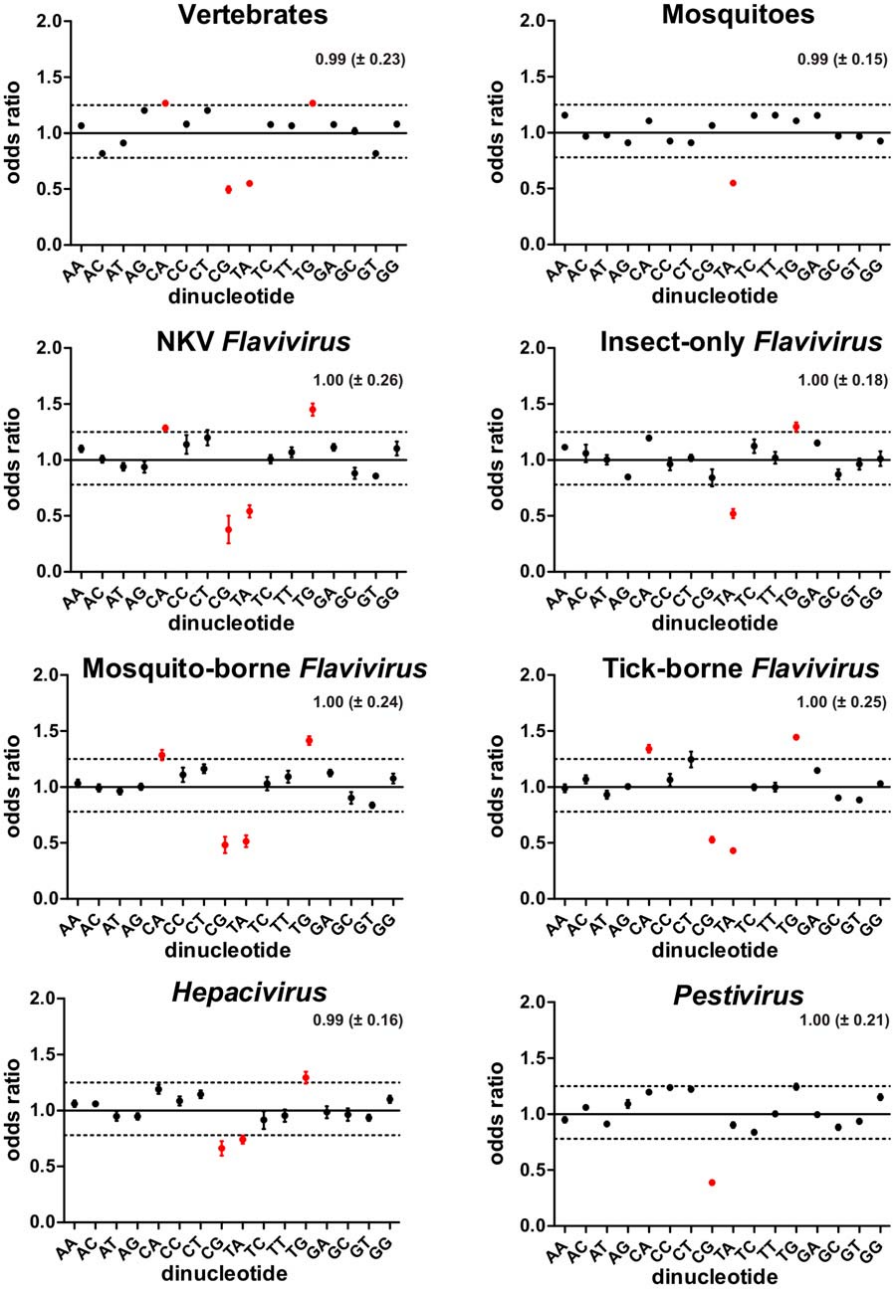
Differential dinucleotide usage in *Flaviviridae* and their hosts. Dinucleotide odds ratio values were calculated for individual species as described in the “Dinucleotide Odds Ratio Calculation” section, and the mean values (±SD) for host and viral groups are plotted. Differentially represented dinucleotides are highlighted in red. Dashed lines indicate cutoffs values of 0.78–1.25. For every group, mean odds ratio values among all dinucleotides (±SD) were calculated and are at the right top in each graph.

Currently, there are no comprehensive phylogenetic studies of the entire *Flaviviridae* virus family in a systematic way. This is certainly due to the immense variability of both genomic and protein sequences within this family. In *Flavivirus* genus, some species are up to 80% distinct when considering their protein sequences [38]. In order to evaluate relatedness-by-speciation relationships among *Flaviviridae* members, we constructed unrooted phylogenetic trees using the entire polyprotein sequence for all *Flaviviridae* viruses by Neighbor-Joining and UPGMA methods, evaluating node confidence values through bootstrapping using 1000 replicates. Both trees obtained displayed the same branching topology and equivalent bootstrap values for all groups defined in this study (data not shown), so we decided to proceed with the resulting Neighbor-Joining tree (Figure 1). A clear division of *Flaviviridae* family into its three known genera can be seen, with *Flavivirus* genus displayed in blue, *Pestivirus* in green and *Hepacivirus*-like in salmon. We decided to analyze GBV-B and GBV-C together with HCV due to their monophyly as a clade ([29] and this study) and to their phenotypical similarity when compared with other *Flaviviridae* [27], [28]. For these reasons, we referred to these viruses as *Hepacivirus* group in this study. *Flavivirus* genus was further divided in four groups to reflect host range and transmission mode, with insect-only *Flavivirus* group highlighted in orange, NKV viruses in purple, mosquito-borne in yellow and tick-borne in red. It is worth noting that, since we were seeking host-induced changes of nucleotide motif usage in viral genomes, the NKV and mosquito-borne *Flavivirus* groups used in this study are paraphyletic. To evaluate possible bias caused by such an approach, these *Flavivirus* groups were further divided to reflect only their evolutionary relatedness, generating a total of seven groups inside *Flavivirus* genus, and dinucleotide odds-ratio analysis was performed in these seven groups as well. Since no major difference was found between this investigation (Figure S1) and the one using the four groups (Figure 2), we decided to keep the two paraphyletic groups to evidence the convergences caused by the NKV and mosquito-borne conditions inside *Flavivirus* genus.

Despite the lack of phylogenetic analyses in *Flaviviridae* family, some studies of this nature had already been done in *Flavivirus* genus, strongly correlating transmission mode to both taxon phylogenetic history and G+C content [39]. *In vitro* experiments also correlated transmission mode to replication ability in distinct host cell types [20]. The viruses groups defined in such studies are in accordance with *Flavivirus* viral clusters defined by our analysis, again evidencing that phylogenetic divisions inside this genus correspond to different transmission modes. Evaluation of ancestral condition for vector presence/absence in *Flavivirus* genus has been, thus far, controversial. Most phylogenetic studies using NS5 sequences strongly suggested that arthropod transmission is a derived trait within genus, being the ancestral condition non-vector transmission [25], [39], [40]. However, Cook and Holmes [41] - which generated three distinct phylogenetic trees by maximum likelihood methods using genes NS3, NS5 and entire genome sequences - suggested that mosquito-borne flaviviruses represent an outgroup to the remaining *Flavivirus* genus members, and tick-borne transmission is a derived trait. These authors also proposed that NKV condition in *Flavivirus* evolved from arboviruses ancestors through several distinct host-loss events. Since several divergent vertebrate-exclusive lineages were found to occur in *Flaviviridae* family (*Pestivirus* and *Hepacivirus* groups and the NKV *Flavivirus* TABV, a presumed vertebrate-exclusive virus currently seen as a putative new *Flaviviridae* genus [24]), it is most parsimonious to assume, based on current data, the ancestral transmission condition in *Flaviviridae* family as non-vector transmission).

### Dinucleotide Composition Analysis

In Figure 2 we summarize mean dinucleotide odds ratios in the host and viral groups. This well-known dinucleotide bias estimator was preferred since its values are usually diverse between species and highly invariant across a given individual genome [42]. Due to these reasons, the dinucleotide odds ratio profile of a genome is often referred as its genomic signature. Besides, odds ratio values are corrected for G+C content and, consequently, dinucleotide biases found using this metric cannot be explained by background nucleotide composition. Each graph also contains the mean (±SD) dinucleotide odds-ratio at its top right position, which we used to estimate the dispersion of dinucleotide odds ratio values for each group. Most dinucleotide usage patterns were located inside a stringent confidence interval already established in specialized literature [43], shown in the graphs as dotted lines. In fact, only four dinucleotides, highlighted in red, were located outside the confidence interval of 0.78–1.25 and were considered differentially represented - CpA, CpG, TpA and TpG (or UpA and UpG for RNA viruses, from now on also referred as TpA and TpG, respectively). When found as differentially represented, CpG and TpA were always under-represented and, conversely, CpA and TpG were always over-represented. Usage biases in these dinucleotides had already been observed within and between genomes in a wide variety of organisms (see, among others, [44]–[47]), and distinct hypotheses have been proposed to account for such observations. These include selection effects [47], mutational bias [48], DNA/RNA structural constraints [49] and combinations of the above.

Among all dinucleotide usage variation found to occur in distinct genomes, perhaps a partial agreement regarding its causes was achieved only for CpG under-representation in vertebrate nuclear genomes. The current working hypothesis to explain it relies on cytosine methylation-deamination model [44]. In brief, it states that vertebrate genomes are subject to cytosine methylation when in the CpG context in order to preserve epigenetic information of a given genomic region, such as gene transcription or silencing [50]. Methylated cytosines are prone to mutate into thymine through spontaneous deamination, generating the dinucleotide TpG with a mismatch pair T/G. This mismatch will, in turn, cause a mutation in the opposite strand if replication occurs without repair, leading to the appearance of the dinucleotide CpA as well [51]. CpG-methylating genomes, such as those of vertebrates, have a global under-representation of CpG dinucleotide and a strong negative correlation between local CpG and TpG abundances. Organisms which do not methylate DNA, such as mosquitoes, displayed no bias for CpG depletion or for TpG and CpA excess [52]. The genomic signature found for both host groups analyzed in this study is in agreement with the literature. Vertebrates displayed a strong under-representation of TpA and CpG, and the over-representation of TpG and CpA as a compensation to CpG avoidance, as expected by their status of CpG-methylating organisms. The only dinucleotide bias found to occur in the insect group was TpA under-representation, coherent with the lack of CpG methylation known to occur in their genomes. Lower than expected frequencies of TpA dinucleotides in coding regions also have a widespread occurrence in genomes regardless of their phylogenetic relationships [53], [54]. Currently two main, non-excluding theories are accepted to explain such bias. The first one states that, since TpA occurs in two out of three stop codons, its avoidance would lead to a lower probability of nonsense mutations. Another theory attributes TpA avoidance in coding regions to its presence in noncoding regulatory motifs such as TATA box and transcription termination regions, thus minimizing the binding of transcription factors to the false initiation sites generated and avoiding spurious transcription [53]. Yet, methylation-deamination model clearly cannot explain CpG under-representation in non-CpG methylated genomes such as mitochondrial and bacterial DNA [46] and several viruses which have only RNA stages during their replication cycle [43].

In order to analyze viral dinucleotide usage patterns and to look for possible correlations between them and the host-specific genomic signatures found to occur, two complementary approaches were adopted. Our first strategy consisted in the comparison of all four *Flavivirus* groups among themselves to minimize phylogenetic distance as a possible noise source, allowing us to check for genomic features putatively induced by host range/type. All vertebrate-infecting *Flavivirus* groups (NKV, mosquito-borne and tick-borne) displayed a dinucleotide usage that mimics the vertebrate genomic signature. CpG and TpA were found to be under-represented and TpG and CpA were over-represented, probably as a compensatory mechanism for CpG avoidance. This genomic signature pattern was already observed in several vertebrate viruses with small genomes, regardless of their genomic material [43], [55]. The methylation-deamination mutation scenario cannot explain CpG depletion and TpG/CpA increase in viruses which possess no intermediate DNA stage through their life cycles. To date, no base methylation event was observed in RNA-only viruses, although methylation in DNA virus is an increasing research field [56]. Recently, an interesting hypothesis of host-induced viral CpG depletion has been suggested for vertebrate RNA viruses based on viral CpG recognition by host immune system [57]. It is well known that CpG, when unmethylated in a DNA context, can induce a strong immunostimulatory response on mammalian immune cells [58]. This is now known to be triggered by the intracellular Pattern Recognition Receptor (PRR) Toll-like 9 (TLR9), which recognizes CpG-unmethylated DNA, preferentially in a T+A rich context, and triggers several immune response pathways [59].Since the vertebrate immune system relies on unmethylated CpG recognition in DNA molecules as a sign of infection, and CpG under-representation in RNA viruses is exclusively observed in vertebrate viruses, it is reasonable to suggest that a TLR9-like mechanism exists in vertebrate immune system which recognizes CpG when in RNA context (such as in the genomes of RNA viruses) and triggers immune responses. An interesting study investigated dinucleotide biases among several RNA viruses, finding strong support for the hypothesis of vertebrate viral CpG depletion induced by host immune system. These authors initially surveyed the G+C genomic content versus CpG odds ratio in RNA viruses which infect several taxa, ranging from bacteria to plants, insects and vertebrates [57]. A strong correlation between these variables was observed only in vertebrate-infecting viruses, indicating a stronger CpG depletion tendency in low G+C content viruses, with no such bias observed in viruses with a high G+C content, where CpG observed odds ratio values are closer to the expected by chance. A very similar CpG depletion tendency was observed in vertebrate genes as well, suggesting that the genomes of RNA vertebrate viruses are selected to mimic some features of host mRNA molecules to avoid immune system detection by an unknown host anti-viral mechanism. Additionally, they found that the influenza A RNA virus, which originated from an avian reservoir and has been infecting human hosts since 1918, was selected under strong pressure to reduce the frequency of CpG in its genome.

The vertebrate immune system-mediated viral CpG depletion hypothesis can account for the CpG depletion observed in vertebrate-infecting *Flaviviridae*. This proposition is further supported by the dinucleotide usage pattern found in insect-only *Flavivirus* group. This cluster also displayed a dinucleotide usage pattern very similar to their host group, having as only biases a TpA decrease and a TpG increase, which can be seen as a putative compensatory mechanism [60] or as a vestigial trait from a putative arbovirus ancestor of insect-only flaviviruses. The absence of CpG depletion in mosquito-only viruses suggests that the viral CpG depletion seen in vertebrate-infecting flaviviruses is induced by their vertebrate hosts. Regarding the TpA avoidance observed in vertebrate-infecting viruses, a TpA recognition system in viral RNA sequences is described as a vertebrate immune response mechanism. As part of dsRNA-activated antiviral pathway, most vertebrates possess a latent intracellular interferon-induced ribonuclease, named Ribonuclease L (RNase L), which degrades double-stranded RNA and activates apoptotic pathways [61], [62]. In *Flaviviridae* family, West Nile Virus (WNV) [63] and *Hepacivirus* [64] are known to be recognized by RNase L, preferentially at UpA or UpU sites [65]. A possible role of insect immune system in the TpA under-representation observed in insect-only viruses is currently an open question, as insect immune response is a largely unexplored, increasing research field. Up to date insect immunity, mainly studied in the model organism *Drosophila melanogaster*, apparently does not rely on motif recognition strategies to detect foreign nucleic acids. RNA interference (RNAi)-mediated immune response appears to be a main factor to deal with a broad range of virus infection [66], [67], as well as innate immune pathways involving Toll receptors [68] and JAK-STAT MAP kinases [69], which also limit viral infection. A recent study correlated the Toll pathway to the control of DENV infection in *Ae. aegypti*, albeit it makes no mention to what virus ligand is responsible for Toll activation [70]. Taken together, *Flavivirus* analysis suggests that a vertebrate-specific evolutionary pressure is actively selecting both host mRNAs and viral genomes against CpG usage and possibly against TpA as well, indirectly leading to the scenario of viral mimicry of host dinucleotide usage. It is worth noting that a TpA decrease also leads to a compensatory increase in TpG, causing its higher over-representation when compared with CpA, since TpG increase is a compensatory mechanism for both CpG and TpA reduction [60]. As predicted by such compensatory mechanism, for all viral groups analyzed in this study, under-representation of both CpG and TpA always led to a higher over-representation of TpG when compared with CpA. The vertebrate group did not display such behavior since it possesses a double-stranded genome, causing CpA and TpG odds ratio to be the same due to Chargaff's parity rule one.

A second strategy to study vertebrate host influence in viral genome composition involved the comparison of the genomic signature of known (*Pestivirus* and *Hepacivirus*) and putative (NKV *Flavivirus*) vertebrate-exclusive *Flaviviridae* groups between them and with the vertebrate genomic signature. By doing so we were able to verify how different evolutionary stories shape dinucleotide usage in viruses which share the same host taxon but are distantly related by phylogeny, thus excluding host variability as a possible source of variation. All these viral groups mimic vertebrate dinucleotide usage to some extent, with some interesting peculiarities in each group. *Pestivirus* genus displayed no TpA bias, a unique feature among all viral groups analyzed. This observation argues against the purifying TpA selection triggered by its presence in regulatory motifs and/or or stop codons. An RNase L-mediated selection in vertebrate-infecting viruses may be occurring if *Pestivirus* members are able to somehow inhibit RNase L or its activation pathway. One quite remarkable characteristic of *Pestivirus* biology is its ability to inhibit the type I interferon (IFN) induction by dsRNA [71], which indirectly inhibits RNase L activation given that the 2–5 oligoadenylate synthetase/RNase L pathway is interferon-dependent [72]. The proteasomal degradation of the Interferon Regulatory Factor 3 (IRF-3) induced by the Npro protein of bovine viral diarrhea virus (BVDV) and classical swine fever virus (CSFV) plays a central role in *Pestivirus* IFN-β pathway inhibition [73], [74]. Furthermore, the surface protein Erns of BDVD also inhibits IFN-β production triggered by dsRNA [75]. In view of such evidences, we consider that *Pestivirus* ability to inhibit RNase L activation is possibly linked to the TpA normal levels found in this group. Nevertheless, any hypothesis to explain TpA usage among *Flaviviridae* must take into account TpA under-representation in insect-only *Flavivirus* as well, since mosquito immune system has no interferon-based antiviral mechanism and no known nucleotide motif recognition signaling pathways. Clearly more experiments must be performed in order to shed some light into this interesting, previously unknown biological phenomenon. *Hepacivirus* group presented the highest level of CpG and TpA frequencies and the lowest level of the putative compensatory TpG usage among all vertebrate-infecting viruses. If we consider such observations in light of the dinucleotide-depletion-by-vertebrate-immune-system hypothesis, viral escape mechanisms are likely to be occurring, as we already proposed for the TpA normal levels observed in *Pestivirus* group. Furthermore, *Hepacivirus* genus is unique among *Flaviviridae* family since their infection commonly evolves to persistence [76]. Yet, due to the absence of an integrative DNA stage in this virus family, such as seen in *Retroviridae* family, a constant production of viral RNA and proteins must occur in *Hepacivirus* members in order to maintain infection persistence. The continuous replication process of HCV is closely associated to the endoplasmic reticulum (ER), since HCV translation and replication take place here [17]. These processes are associated to a huge production of viral proteins into the ER, which disrupts its normal functions and induces ER stress [77]. Consequently, a cellular adaptive response, named unfolded protein response (UPR), is activated to diminish this stress via unfolded protein degradation, stimulation of protein folding, down-regulation of protein synthesis or apoptosis induction [78], [79]. In order to maintain persistence, HCV is able to suppress UPR by inhibiting several nodes of UPR activating pathways [79]. Therefore, HCV inhibition of UPR allows HCV viral replication and its persistence in infected cells. Additionally, a continuous viral production leads to an increasing chance of detection by host immune system, and viral escape mechanisms are likely to be positively selected. In fact, *Hepacivirus* genus is known for its unique ability among *Flaviviridae* to modulate both innate and adaptive host immune system [80] as well as to mimic host proteins [81], [82] to avoid host immune system detection. For us it seems likely that the quasi-normal CpG and TpA frequencies in *Hepacivirus* genomic signature is a direct consequence of the viral escape mechanisms that were selected due to its persistence. This genus also possesses the highest G+C content among all viral groups surveyed (data not shown), a fact which also allows a higher CpG content according to the host-induced viral CpG depletion hypothesis [57]. Dinucleotide odds ratio SD values (represented in the top-right top position of each graph) allowed the division of viral groups into two clusters. *Hepacivirus* and insect-only *Flavivirus* dinucleotide odds ratio values deviate less from expectation than the other viral groups, indicating that these viral groups are using dinucleotides at a frequency closer to the expected usage values.

### Codon Usage Similarity

**Figure 3:**
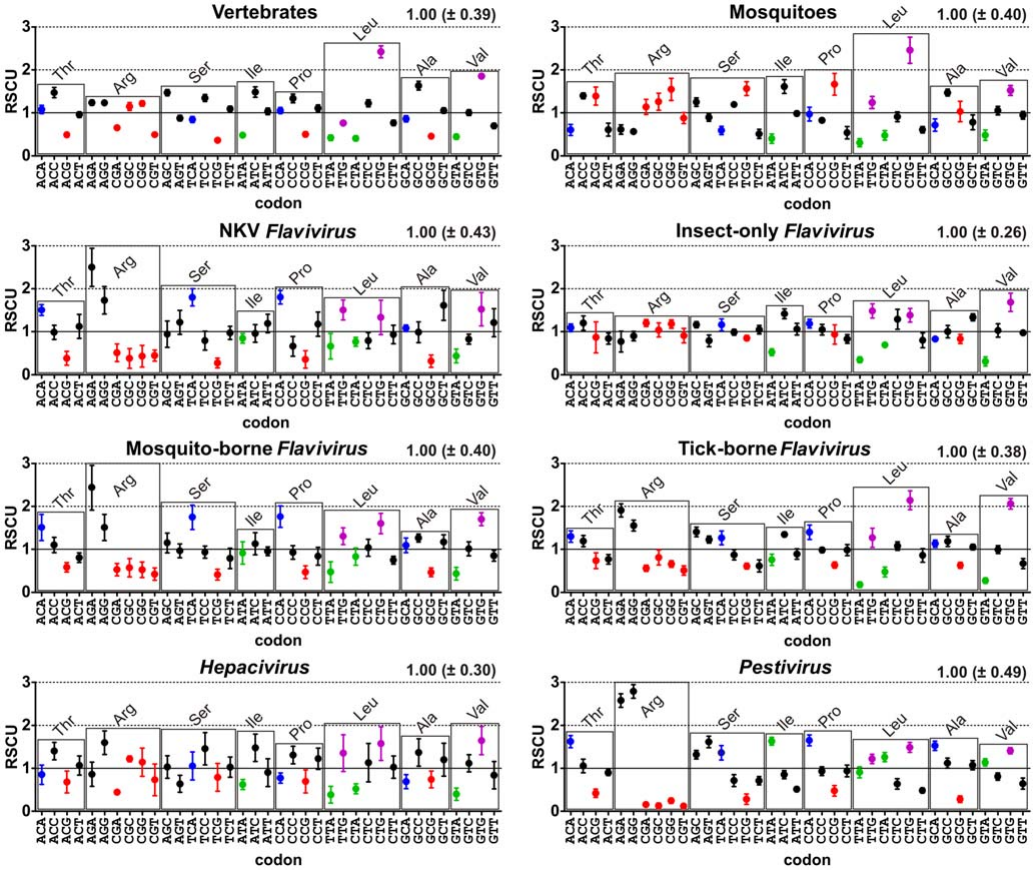
Relative Synonymous Codon Usage (RSCU) in *Flaviviridae* and their hosts. RSCU mean values (±SD) were calculated for each host and viral group as described in the “Relative Synonymous Codon Usage Calculation” section. Only groups of synonymous codon containing one codon with a dinucleotide found as differentially represented (CpG, TpA, TpG or CpA) and one codon without them are displayed to allow relative comparison. Each box represents a set of synonymous codons possessing such property. CpG-containing codons are highlighted in red, TpA-containing are in green, TpG-containing are in purple and CpA-containing are in blue. For every group, mean RSCU values among all codons (±SD) were calculated and are at the right top in each graph.

To verify if the viral mimicry of the host-specific genomic signatures influences synonymous codon usage preferences in viral genomes, we also searched for possible codon usage biases in these groups. We calculated mean (±SD) RSCU values for all groups (as described in “Methods” section) and plotted the values found for every informative synonymous codon (Figure 3). Informative synonymous codons were defined as any trinucleotide containing a dinucleotide found as differentially represented in the dinucleotide analysis (CpG, TpA, CpA or TpG) and at least one synonymous codon without the aforementioned dinucleotides to allow relative comparison. Examples of non-informative codons are TAC-TAT, TGT-TGC, CAT-CAC and CAA-CAG pairs, since both codons in each pair contain a differentially represented dinucleotide (TpA, TpG, CpA) and code for a dicodonic amino acid. Informative CpG-containing codons are highlighted in red, TpA-containing ones are in green, TpG-containing ones are in purple and CpA-containing are in blue. Synonymous codons are grouped by boxes corresponding to amino acids to allow codon usage comparison excluding amino acid frequency biases as a source of variation. We initially observed that all group-specific dinucleotide biases found in this study are deeply reflected in their choices for synonymous codon usage. The vertebrate group displayed a strong tendency to avoid CpG containing codons, with six out of eight of them less used than expected. Vertebrate-infecting viral groups from *Flavivirus* genus (NKV, mosquito-borne and tick-borne) were found to avoid CpG-containing codons as well, since none of these codons was observed with a RSCU value above one. This observation was further supported by the analysis of tetracodonic amino acids (threonine (Thr), proline (Pro), alanine (Ala), valine (Val) and serine (Ser) TCN codons) where, for both vertebrates and vertebrate-infecting *Flavivirus*, CpG-containing codons were clearly less used than the CpA-containing codons. The invertebrate host group displayed no tendency for CpG under-representation being, instead, the only taxon where CpG-containing codons were used more than CpA-containing codons for all tetracodonic amino acids. Insect-only *Flavivirus* also mimicked its host group in synonymous codon usage preferences, with both groups displaying as main bias the avoidance of TpA-containing codons. This viral group also displayed a much less pronounced bias for CpG under-representation when compared to the other viral groups (except *Hepacivirus*, see below), having the lowest difference between CpG and CpA-containing codons in tetracodonic amino acid analysis. The analysis of tetracodonic amino acid boxes in *Hepacivirus* group revealed almost no difference between informative codons, which is coherent with the scarcely noticeable CpG and TpA under-representation found in the dinucleotide analysis. These observations again suggest that HCV immune system escape mechanisms could be key factors to understand its nucleotide motif usage patterns. *Pestivirus* group displayed a strong CpG reduction bias, having the lowest RSCU values for CpG-containing codons among all groups. TpA usage pattern in this group also displayed the same intriguing phenomenon observed in dinucleotide analysis. This viral group was the only one possessing TpA-containing informative codons with RSCU values above one (Figure 3, Isoleucine (Ile), valine (Val) and leucine (Leu) TTR and CTN codons). Although TpA codons were always more used than expected in Ile, Val and Leu boxes (with the exception of TTA Leu codon), G-ending codons were always preferred to A-ending ones in Leu TTR and Val CTN codons, probably due to a compensatory TpG increase caused by the extreme CpG under-representation found in this virus group [60]. TpG over-representation, a compensatory mechanism for both TpA and CpG biases, was spread across all groups surveyed, as can be seen by the informative TpG-containing codons, highlighted in purple. RSCU mean value (±SD) (represented in the top-right top position of each graph) once more allowed the division of viral groups in two clusters, the same found in the dinucleotide odds ratio SD analysis. *Hepacivirus* and insect-only *Flavivirus* displayed a much less biased codon usage profile, again suggesting that the pressures which shape nucleotide motif usage in these groups are less effective when compared to the other viral groups.

**Figure 4:**
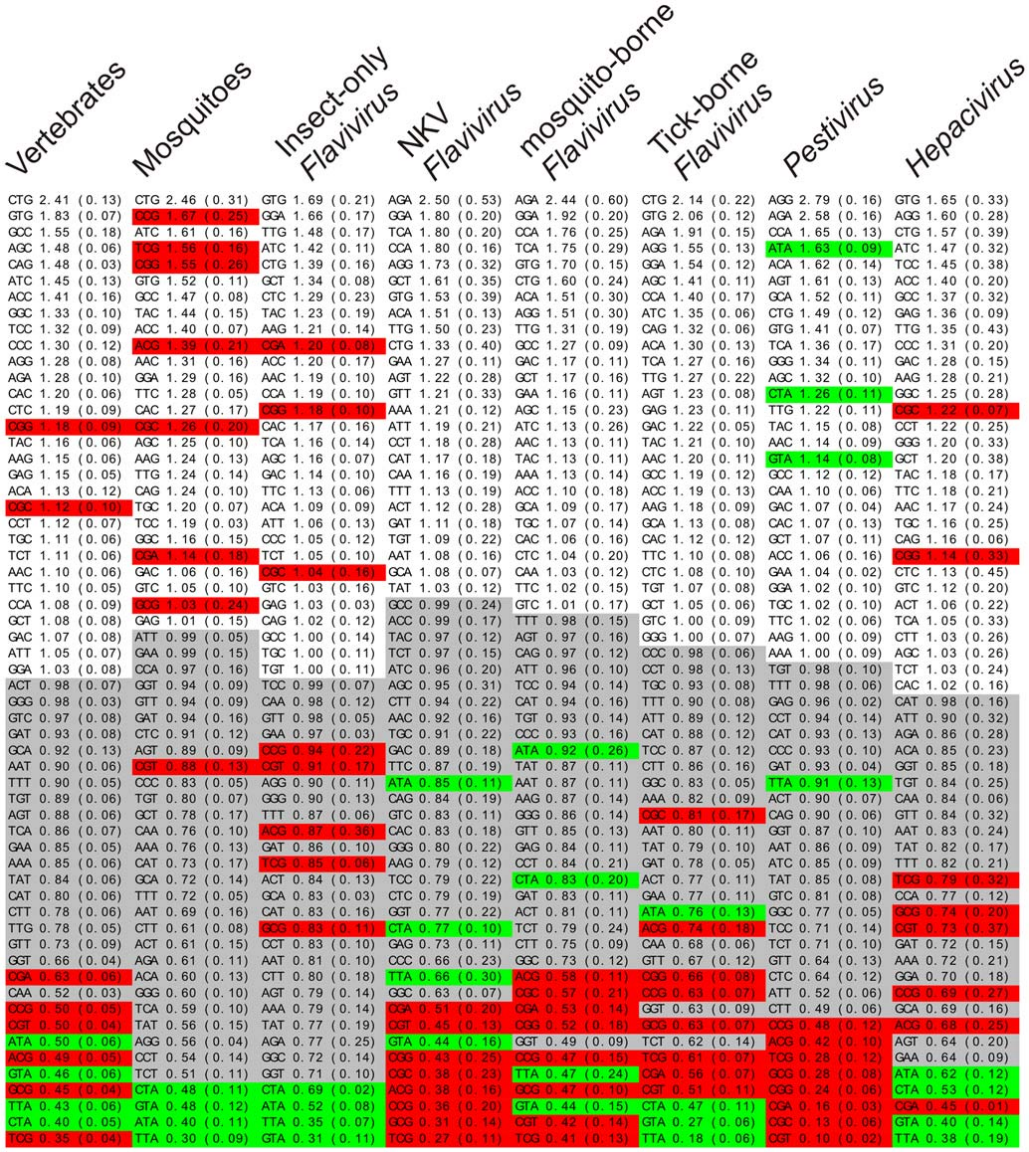
Ordered list of codons in *Flaviviridae* and hosts groups by their mean RSCU values. Mean RSCU values were calculated for each host and viral group as defined in the “Relative Synonymous Codon Usage Calculation” section. The codons in each group are ordered from low to top by their mean RSCU values. CpG-containing codons are highlighted in red and TpA-containing are in green. Codons with RSCU values smaller than one are highlighted in grey.

**Figure 5:**
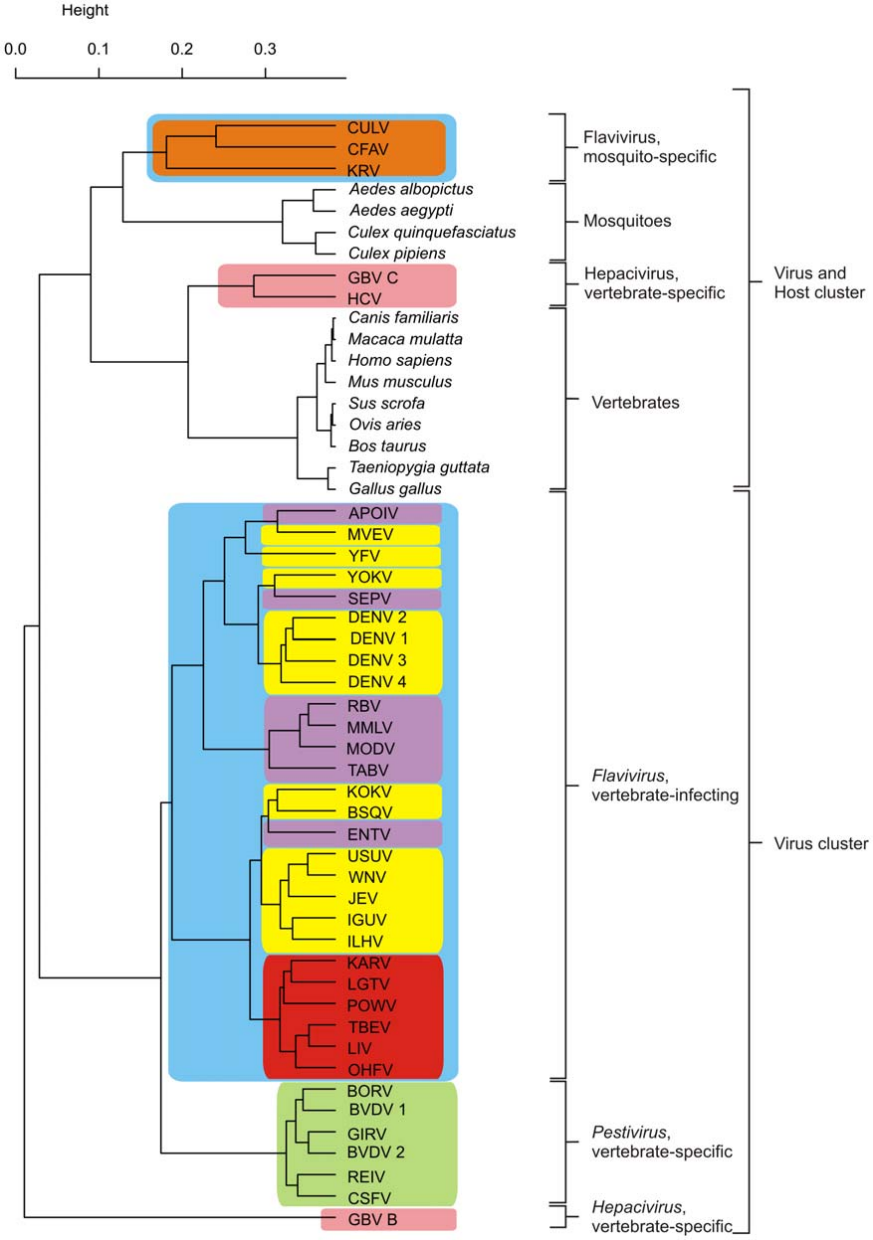
Hierarchical clustering of *Flaviviridae* viruses and their hosts based on codon usage likeness. RSCU values were calculated for codons with degeneracy greater than one for every organism, generating a matrix of N×M dimensions, being N the number of species and M the number of degenerated codons. Hierarchical clustering of organisms was conducted based on Spearman's rho correlation coefficients of RSCU values. Color schema for clusters is the same used in Figure 1. *Flavivirus* genus is highlighted in blue, *Pestivirus* is in green and *Hepacivirus* is in salmon. *Flavivirus* taxon was further divided according to host range: orange - insect-only, purple - NKV, yellow - mosquito-borne, red - tick-borne. Acronyms and names of viruses are defined in the “viral dataset” section.

To further evidence the roles of CpG and TpA dinucleotides in codon usage preference, we also generate an ordered list of codons for every group by their mean RSCU values (Figure 4). Color scheme evidences informative CpG- and TpA-containing codons in red and green, respectively. Grey boxes indicate codons with RSCU values below one. A general tendency for under-representation of CpG-containing codons was observed in the vertebrate group as well as in all the viruses which infect them. These codons comprise the vast majority of the less used codons in these groups, suggesting that dinucleotide biases influences synonymous codon usage choice in these organisms. The overall similarity between the mosquito group and the insect-only *Flavivirus* was also evident, with both groups displaying a strong TpA under-representation bias and, apparently, no CpG bias at all, since CpG-containing codons were found spread across the list with no discernible usage pattern. The peculiarities found in the previous analyses of dinucleotide and synonymous codon usage preferences in the mammal-exclusive *Pestivirus* and *Hepacivirus* groups are, again, evident. *Pestivirus* genus presented the strongest CpG under-representation bias among all groups surveyed, with the eight less used codons containing the CpG dinucleotide. This virus group was also the only one possessing TpA-containing codons with usage values above one. *Hepacivirus* mimicry behavior of vertebrate host is even more evident now, since they share the same subset of CpG-informative codons over (CGC and CGG) and under-represented (TCG, GCG, CGT, CCG, ACG and CGA). In fact, visual inspection suggests a very similar codon usage pattern between the viral groups of insect-only *Flavivirus* and *Hepacivirus* and their respective host groups, which was further evidenced by hierarchical clustering analysis based on codon usage (Figure 5).

As a final analysis of codon usage likeness, we investigated individual codon usage among all species used in this study in an attempt to evaluate general codon usage similarities among them. This analysis was done by performing hierarchical clustering of all species in this study by their codon usage similarity, using Spearman's rank correlation as a similarity measure (Figure 5). Color schema is the same as Figure 1 for comparison purposes. *Flavivirus* genus is highlighted in blue, *Pestivirus* in green and *Hepacivirus* group in salmon. *Flavivirus* genus was further divided into insect-only *Flavivirus* group highlighted in orange, NKV in purple, mosquito-borne in yellow and tick-borne in red. Three main groups were observed in this analysis. One cluster contains the vast majority of viruses used in this study, which are roughly grouped by their phylogenetic relatedness. *Flavivirus* vertebrate-infecting members are all grouped together, with some internal discrepancies between phylogenetic-based and codon-based clustering. This observation suggests that a common codon usage signature exists for vertebrate-infecting *Flavivirus* species. However, it appears that the codon signature is not able to resolve phylogenetic relationships beyond the genus level in this taxon. With the exception of some conserved clusters, such as the tick-borne *Flavivirus* and the DENV group, no clear correlation was found between the internal branches of codon-based and phylogeny-based clustering. *Pestivirus* genus also clustered as a separate group in codon analysis, again suggesting that codon usage signatures contain phylogenetic information in *Flaviviridae* viruses. The unique codon usage signature seen in this genus is probably due to its already seen unusual codon usage pattern when compared to the other viral groups (Figures 3 and 4). The second cluster observed contained all hosts, which clustered in two subgroups corresponding to the vertebrate and invertebrate groups used in this study. This grouping pattern suggests that a common, host-specific signature for codon usage exist, which allowed a clear distinction between host and viral groups. The clustering patterns inside each host group also reflected their known phylogeny. In the vertebrate taxon, a clear division between birds and mammals was observed. The *mammalia* taxon was further divided into other monophyletic taxa. The organisms *B. taurus*, *O. Aries* and *S. Scrofa* belong to the *Artiodactyla* order. The other cluster (*M. musculus*, *H. sapiens*, *M. mulatta* and *C. familiaris*) correspond to members of the mammalian clade *Boreoeutheria*. Nevertheless, discrepancies between phylogenetical and codon usage trees were observed, such as the closest codon usage similarity between *M. mulatta* (primate) and *C. Familiaris* (carnivora) when compared to *H. sapiens* (primate). Furthermore, *C. familialirs* belongs to the *Carnivora* order, a taxon which is more related to the *Artiodactyla* order than to the *Euarchontoglires* superorder, which is composed of rodents and primates [83]. These observations again suggest that phylogenetic information contained in codon usage similarity values is better appropriated to classify more distant groups. The invertebrate host group also presented internal grouping patterns coherent with their phylogenetical relationships, since the *Culex* and *Aedes* genus members grouped together. Of special interest in the second cluster was the finding of single-host viruses from *Hepacivirus* and insect-only *Flavivirus* groups that clustered together with their corresponding host group. This finding suggests that these viral groups, distantly related when comparing their protein sequences (Figure 1), convergently changed their nucleotide content in response to a common, host-induced factor, which shaped dinucleotide and codon usage in these viruses and their hosts. Adaptation to host-specific pressures apparently occurred as well, since these viral groups specifically grouped with their corresponding hosts groups. A possible reason for this unexpected, intriguing finding is the fact that these viruses are the only known in *Flaviviridae* family where infection can evolve to persistence. *Hepacivirus* chronicity is a well-known trait [17], and insect-only flaviviruses were found infecting mosquito pupae and apparently are able to keep infection through their entire lifespan [22]. As already seen, these virus groups were also the ones with the less biased nucleotide motif usage profile, as seen by their lower SD values for mean dinucleotide odds ratio and mean RSCU values (Figures 2 and 3). In the light of these facts, we hypothesize that *Hepacivirus* and insect-only *Flavivirus* infection persistence led to the emergence of distinct viral escape mechanisms in both groups to mimicry host-specific codon usage patterns. Since *Flaviviridae* do not posses a DNA intermediate stage, a continuous virion production is needed in order to keep infection. Thus, these viral groups are expected to developed mechanisms to avoid or to decrease their recognition by host immune system during their long infection periods. The reduction of host-induced pressures, which are at least in part responsible for the distortion in viral dinucleotide and codon usage preferences, is likely to have led to the quasi-normal nucleotide motif usage seen in *Hepacivirus* and insect-only *Flavivirus*. A single *Hepacivirus* member, HGB-B, formed a separated cluster, suggesting that an ongoing adaptation process to the vertebrate codon usage signature may be happening. In fact, among all viruses from *Hepacivirus* group used in this study, GB-B is the only one recovered from a New World monkey, being the other two of human origin [27].

*Flaviviridae*-host genome coevolution is mediated by a rich and complex sum of factors caused by several coadaptational events in both organisms which, acting on several levels, such as the dinucleotide and codon structures, convergently shaped nucleotide motif usage in such contrasting groups. We propose that the common patterns of nucleotide motif usage observed in hosts and viruses coding sequences are caused by host-induced and host-specific constraints, and are key factors in understanding the genomic evolution of *Flaviviridae* family and its hosts.

## Methods

Perl and R [84] scripts were used to analyze most of the data in this study and are available from the authors upon request. Usage of third-party software is mentioned when appropriate.

### Data Acquisition

All files used in this study were downloaded from the National Center for Biotechnology Information (NCBI) website in GenBank format using RefSeq [85] data when available or GenBank [86] otherwise. Additional information regarding each dataset, such as species composition and number of sequences is available at Table S1.

### Datasets

#### Host Dataset

The host dataset consists of the entire set of complete mRNA sequences from different species of *Vertebrata* and *Culicidae* taxa that, after extensive literary research, are known to be infected by *Flaviviridae* viruses. *Vertebrata* dataset was composed of species *Gallus gallus* (chicken), *Taeniopygia guttata* (zebra finch), *Homo sapiens* (man), *Sus scrofa* (pig), *Bos taurus* (cow), *Canis familiaris* (dog), *Macaca mulatta* (Rhesus monkey), *Mus musculus* (mouse) and *Ovis aries* (sheep). *Culicidae* data was composed of species *Culex quinquefasciatus*, *C. pipiens*, *Aedes aegypti* and *Ae. albopictus*. In order to study specific genomic signatures of each host taxa the eukaryotic dataset was further divided into vertebrate and invertebrate groups.

#### Viral Dataset

All complete genome sequences from *Flaviviridae* virus family used in this study were obtained by an initial query into NCBI genome database for complete genomic sequences from *Flaviviridae* family using Entrez [87]. This query returned a single, reference sequence of each species within this taxon. The reference sequence of a given genome in this database comprises the first complete sequence obtained for it. In order to study specific genomic features caused by speciation and/or by different host infection range, the viral dataset was further divided into six distinct, non-redundant groups based on host specificity and phylogenetic relatedness (see subsection “*Flaviviridae* phylogenetic analysis” below for details). *Flavivirus* genus was divided into four datasets - non known arthropod vector (NKV) *Flavivirus*, insect-only *Flavivirus*, mosquito-borne *Flavivirus* and tick-borne *Flavivirus* - and the two remaining genera (*Hepacivirus* and *Pestivirus*) were considered as two distinct vertebrate-exclusive groups. Viral sample names and acronyms used through this article are as follows: Apoi virus (APOIV), Border disease virus (BORV), Bovine viral diarrhea virus 1 (BVDV 1), Bovine viral diarrhea virus 2 (BVDV 2), Bussuquara virus (BSQV), Cell fusing agent virus (CFAV), Classical swine fever virus (CSFV), Culex *Flavivirus* (CULV), Dengue type 1 virus (DENV 1), Dengue type 2 virus (DENV 2), Dengue type 3 virus (DENV 3), Dengue type 4 virus (DENV 4), Entebbe bat virus (ENTV), GB-B virus (GBV-B), GB-C virus (GBV-C), Giraffe *Pestivirus* (GIRV 1), Hepatitis C virus (HCV), Iguape virus (IGUV), Ilheus virus (ILHV), Japanese encephalitis virus (JEV), Kamiti River virus (KRV), Karshi virus (KARV), Kokobera virus (KOKV), Langat virus (LGTV), Louping ill virus (LIV), Modoc virus (MODV), Montana myotis leukoencephalitis virus (MMLV), Murray valley encephalitis virus (MVEV), Omsk hemorrhagic fever virus (OHFV), Powassan virus (POWV), Reindeer 1 *Pestivirus* (REIV), Rio bravo virus (RBV), Sepik virus (SEPV), Tamana bat virus (TABV), Tick-borne encephalitis virus (TBEV), Usutu virus (USUV), West nile virus (WNV), Yellow fever virus (YFV), Yokose virus (YOKV)

### Data Treatment

Perl scripts were used to extract the open reading frames (ORFs) from the GenBank files downloaded from NCBI, as described in “Data Acquisition” Section. All ORFs were submitted to the following cleaning pipeline to remove possible error sources. In order to be considered as a valid gene (or a valid genome in the case of viruses, since they possess a single known ORF), a given ORF should not be classified as a hypothetical product, start with a valid start codon, end with a valid stop codon, have a length multiple of three, and have no ambiguous bases, here considered as a non-standard (A, C, T or G) nucleotide. Since different nucleotide bias are known to occur in nuclear and mitochondrial genomes [46], only nuclear genes were analyzed in eukaryotic genomes.

### Virus Phylogenetic Analysis

The complete polyprotein sequences of each *Flaviviridae* member with a valid genome as defined by “Data treatment” section was aligned using CLUSTALW software with default parameters [88]. The resulting alignment file was used to construct a phylogenetic tree using MEGA [89] software with default parameters. Trees were generated using both Neighbor-Joining and UPGMA algorithms. To evaluate node confidence values, bootstrap analysis were performed using MEGA default parameters and 1000 replicates.

### Dinucleotide Odds Ratio Calculation

A common assessment of dinucleotide bias in sequences is the odds ratio, defined as the quotient of the probability of finding a dinucleotide in a given sequence divided by the product of the probabilities of finding each nucleotide that forms the pair in the same sequence, calculated as shown in Equation 1.

Equation 1 – Calculation of dinucleotide odds ratio
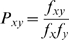
Where 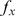 and 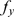 denote the frequency of mononucleotides x and y in a given sequence and 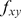 denotes the frequency of dinucleotide *xy* in the same sequence. As a very conservative criterion, dinucleotides with odds ratio values outside the 0.78–1.25 range were considered as having a low or high relative abundance, respectively [43]. This calculation is valid only for single stranded sequences, which do not obey Chargaff's parity rule one. In the case of organisms with double-stranded genomic material, the frequency of each dinucleotide must be calculated in a symmetric manner, also considering the opposite strand. Thus, if we denote the symmetric frequency by an “°” mark, we have f°(T)  =  f°(A)  =  (f(A) + f(T))/2 and f°(C)  =  f°(G)  =  (f(C) + f(G))/2 and, by consequence, f°(AC)  =  f°(GT)  =  (f(AC) + f(GT))/2. As a result of Chargaff's parity rule one and Equation 1 we calculated the symmetric odds ratio as shown in Equation 2.

Equation 2 - Calculation of symmetrical dinucleotide odds ratio
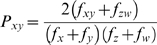
Where *x* and *y* denote two dinucleotides, and *z* and *w* denote the two complementary nucleotides of *y* and *x*, respectively. As an example, if *x* and *y* represents the nucleotides A and C, calculation was done as follows: P°(AC)  =  P°(GT)  =  2(f(AC) + f(GT))/(f(A)+f(T))(f(C)+f(G)).

Dinucleotide odds ratio for *Flaviviridae* members were calculated using odds ratio and hosts dinucleotide odds ratio were calculated using symmetric odds ratio.

### Relative Synonymous Codon Usage Calculation

Relative Synonymous Codon Usage (RSCU) is a common method used to estimate codon bias for all codons which code for an amino acid with degeneracy greater than one. It is defined as the observed frequency of a codon *j* in a sequence *x* divided by the frequency expected *E* if all synonymous codons for the amino acid coded by *j* were equally frequent, as shown in Equation 3.

Equation 3 – Calculation of RSCU
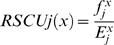
Where 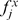 is the observed frequency of codon *j* in genome *x* and 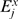 is the expected frequency of the codon *j*. Expected values are calculated by counting the total number of synonymous codons for a given amino acid in the sequence divided by the number of existing codons that codes for it.

### Correlation Analysis of Codon Bias in Different Organisms

For each organism listed in Table S1 we calculated RSCU for every codon that codes for an amino acid with degeneracy greater than one (i.e., monocodonic amino acids and stop codons were not used), which generated a 59-dimension codon vector for every organism. These codon vectors were organized in a matrix of *N*×*M* dimensions, *N* being the number of species and *M* the number of degenerated codons. Hierarchical clustering of this matrix was conducted based on Spearman's rho correlation coefficients of RSCU values, conceptually similar as done by [90].

### Percentage of Dinucleotide and Codon Usage Deviation

To evaluate percentage of deviation in dinucleotide and codon frequencies from the mean values, we performed the calculation of mean and standard deviation values of dinucleotide odds-ratio and RSCU measurements for host and viral groups. The main underlying idea of this analysis is that organisms subject to less constraint factors for usage of a given dinucleotide/codon should deviate from the mean value less than highly biased organisms.

## Supporting Information

Table S1 This table contains all species used in this study.
(0.03 MB XLS)

Figure S1 Dinucleotide odds ratio analysis of monophyletic Flavivirus groups.
(0.56 MB TIF)
